# [Corrigendum] Bax inhibitor-1 suppresses early brain injury following experimental subarachnoid hemorrhage in rats

**DOI:** 10.3892/ijmm.2024.5362

**Published:** 2024-02-28

**Authors:** Jiaxin Liu, Shuai Zhou, Yueting Zhang, Xiuying Li, Xiying Qian, Weihua Tao, Lide Jin, Jianhua Zhao

Int J Mol Med 42: 2891-2902, 2018; DOI: 10.3892/ijmm.2018.3858

Following the publication of the above article, an interested reader drew to the authors' attention that, in Fig. 6 on p. 2898, the 'SAH' and 'SAH+NC' data panels contained an apparently overlapping section of data, such that these data appeared to have been derived from the same original source, even though they were intended to show the results from differently performed experiments.

The authors have examined their original data, and realize that the 'SAH+NC' data panel had inadvertently been selected incorrectly for this figure. In addition, in response to a further query from the reader, the authors wished to point out that the standard deviations in their study were statistically analysed using GraphPad Prism software version 5.0a.

The revised version of Fig. 6, now showing the correct data for the 'SAH+NC' experiment, is shown on the next page. The authors can confirm that the errors associated with this figure did not have any significant impact on either the results or the conclusions reported in this study, and all the authors agree with the publication of this Corrigendum. The authors are grateful to the Editor of *International Journal of Molecular Medicine* for allowing them the opportunity to publish this Corrigendum; furthermore, they apologize to the readership of the Journal for any inconvenience caused.

## Figures and Tables

**Figure 6:**
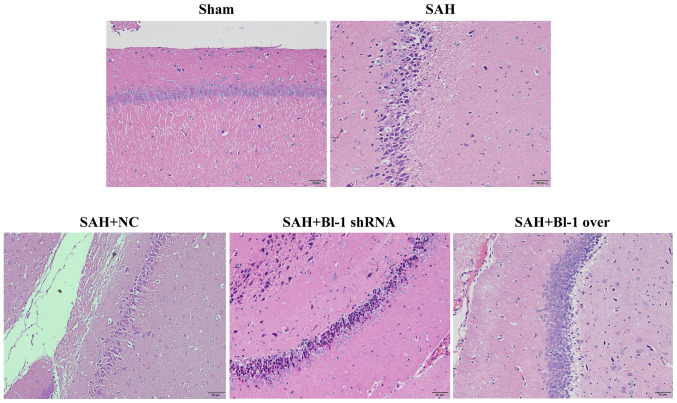
H&E staining of rats following BI-1 overexpression and shRNA silencing. Normal and heteromorphic neurons in the hippocampus of the brains were detected by H&E staining in the sham, SAH, SAH+NC, SAH+BI-1 shRNA and SAH+BI-1 over groups. Scale bar=50 *μ*m (magnification, ×200). H&E, hematoxylin and eosin; BI-1, B-cell lymphoma 2-associated X protein-inhibitor-1; shRNA, short hairpin RNA; SAH, subarachnoid hemorrhage; SAH+BI-1 over, SAH + BI-1 overexpression; NC, negative control.

